# Supplementary figures and images for: Apolipoprotein E regulates the maturation of injury-induced adult-born hippocampal neurons following traumatic brain injury
Source: PLoS One. 2020 Mar 2;15(3):e0229240. doi: 10.1371/journal.pone.0229240 (PMC7051085; doi:10.1371/journal.pone.0229240)

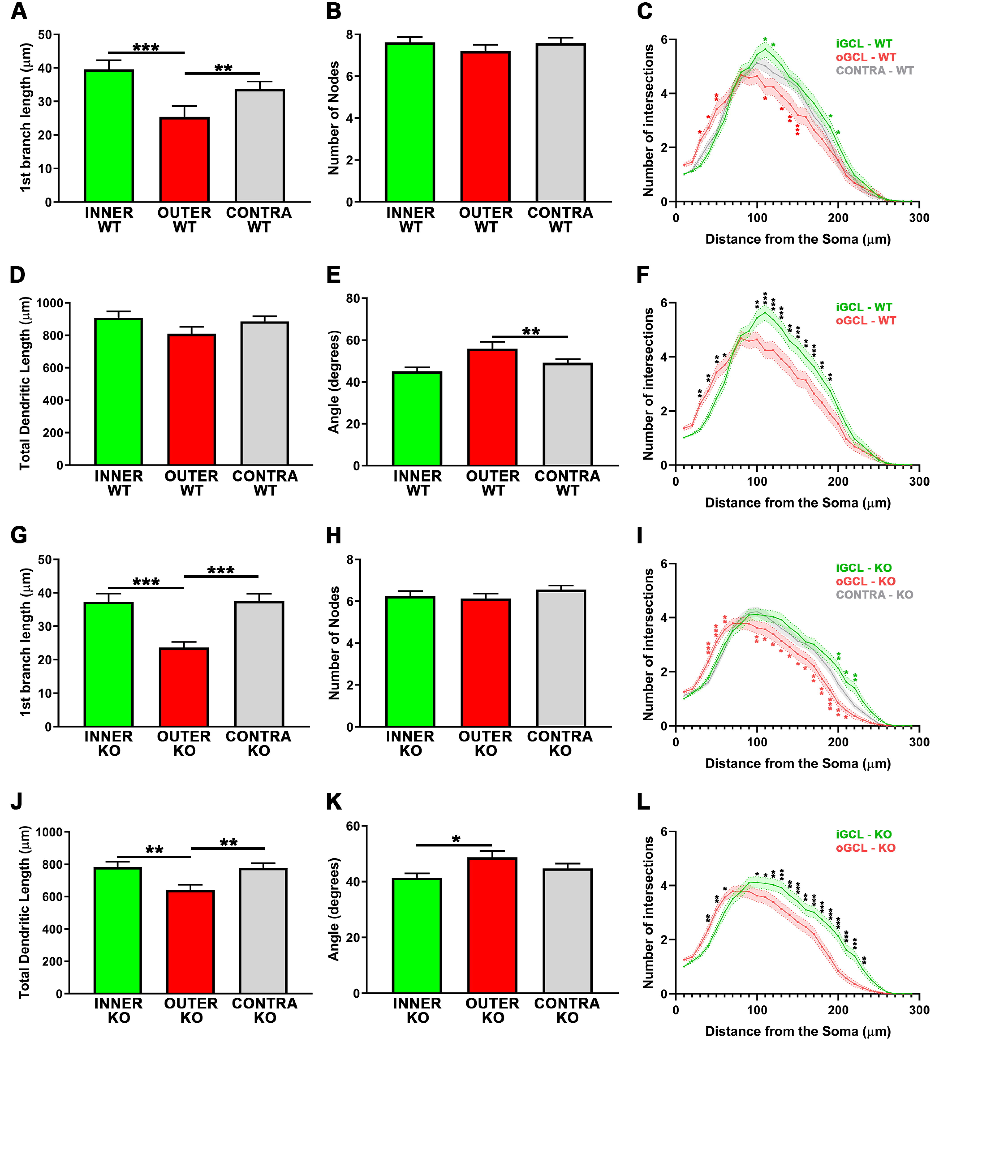

Supplement: S1 Fig — WT injury-induced adult-born granule neurons found in the oGCL branched closer to their cell body when compared to adult-born granule neurons found in the iGCL (p < 0.001) or contralateral (p < 0.01) dentate gyrus (A) while the number of dendritic divisions is similar between adult-born granule neurons independently of their soma localization (B). (C) Sholl analysis exposed significant differences in dendritic branching observed in both proximal and distal regions when comparing the oGCL with the Contralateral side (red stars) and in more distal regions when comparing iGCL with Contralateral side (green stars). (D) The cumulative dendritic length is similar in the three conditions. (E) Injury-induced adult-born granule neurons found in the oGCL have a wider dendritic span when compared to matching cells from the contralateral side (p < 0.05) but not the iGCL. (F) Sholl analysis also revealed differences in the dendritic patterns of cells found in the iGCL with the oGCL in both proximal and distal regions. 4 mice/condition and at least 10 neurons/mouse were analyzed; iGCL: 61 cells; oGCL: 45 cells; Contra: 60 cells. ApoE KO injury-induced adult-born granule neurons found in the oGCL branched closer to their cell body when compared to adult-born granule neurons found in the iGCL (p < 0.001) or contralateral (p < 0.001) dentate gyrus (G) while the number of dendritic divisions is similar between adult-born granule neurons independently of their soma localization (H). (I) Sholl analysis exposed significant differences in dendritic branching observed in both proximal and distal regions when comparing the oGCL with the Contralateral side (red stars) and in more distal regions when comparing iGCL with Contralateral side (green stars). (J) The cumulative dendritic length was decreased in neurons found in the oGCL when compared to iGCL or Contralateral side (p < 0.001). (K) Injury-induced adult-born granule neurons found in the oGCL have a wider dendritic span when compared [file pone.0229240.s001.tif]

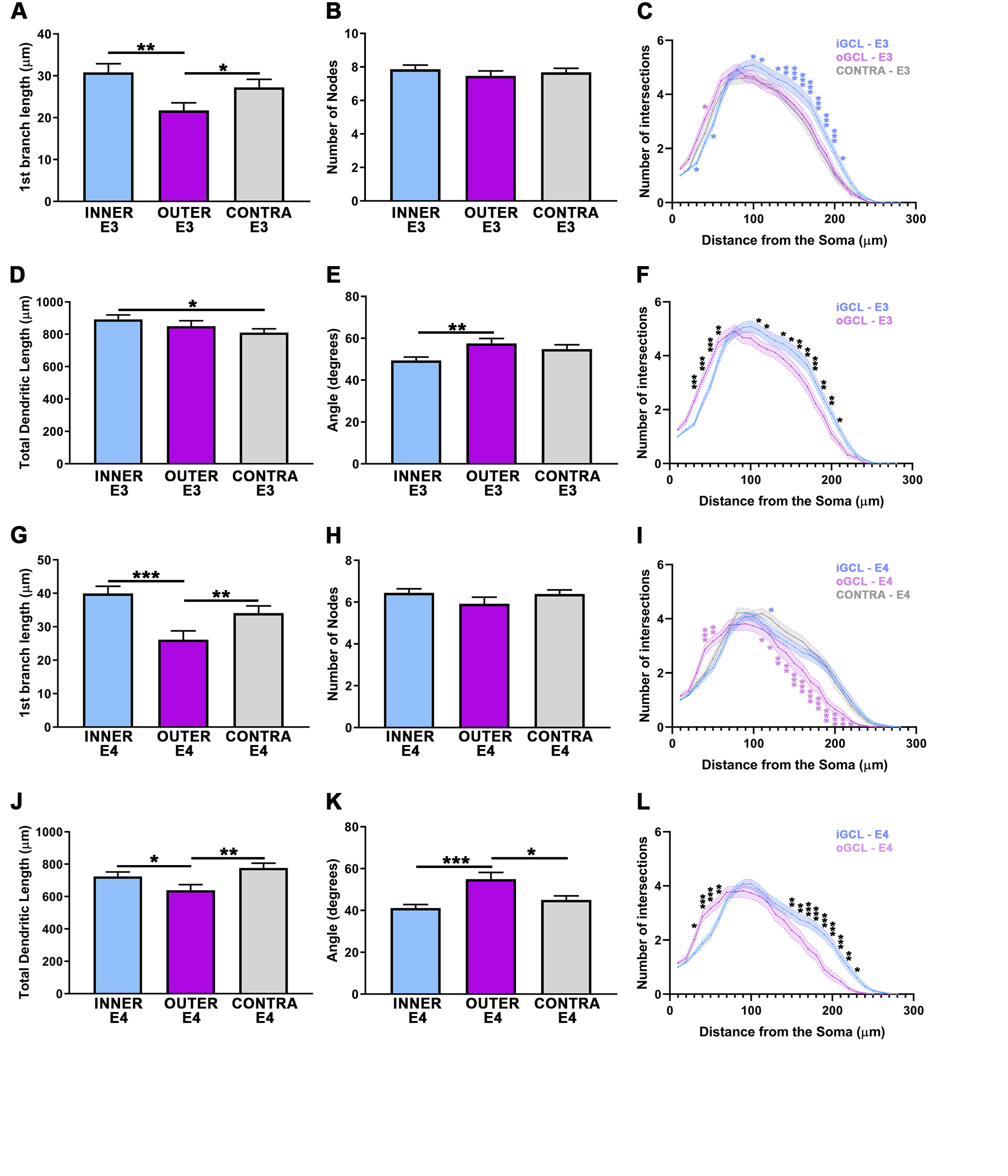

Supplement: S2 Fig — ApoE3 injury-induced adult-born granule neurons found in the oGCL branched closer to their cell body when compared to adult-born granule neurons found in the iGCL (p < 0.01) or contralateral (p < 0.05) dentate gyrus (A) while the number of dendritic divisions is similar between adult-born granule neurons independently of their soma localization (B). (C) Sholl analysis exposed significant differences in dendritic branching observed in both proximal and distal regions when comparing the iGCL with the contralateral side (blue stars) and minor differences in proximal regions when comparing oGCL with contralateral side (purple stars). (D) The cumulative dendritic length in the contralateral side was reduced when compared to iGCL (p < 0.05). (E) Injury-induced adult-born granule neurons found in the oGCL have a wider dendritic span when compared to matching cells from the iGCL (p < 0.05) but not the contralateral side. (F) Sholl analysis also revealed differences in the dendritic patterns of cells found in the iGCL with the oGCL in both proximal and distal regions. 4 mice/condition and at least 10 neurons/mouse were analyzed; iGCL: 81 cells; oGCL: 62 cells; Contra: 81 cells. ApoE4 injury-induced adult-born granule neurons found in the oGCL branched closer to their cell body when compared to adult-born granule neurons found in the iGCL (p < 0.001) or contralateral (p < 0.01) dentate gyrus (G) while the number of dendritic divisions is similar between adult-born granule neurons independently of their soma localization (H). (I) Sholl analysis exposed significant differences in dendritic branching observed in both proximal and distal regions when comparing the oGCL with the Contralateral side (purple stars) while minor differences have been found when comparing iGCL with Contralateral side (blue stars). (J) The cumulative dendritic length was decreased in neurons found in the oGCL when compared to iGCL (p < 0.05) or contralateral side (p < 0.01). (K) Injury-induced adult-born [file pone.0229240.s002.tif]
