# Supplementary material for: Apolipoprotein E regulates the maturation of injury-induced adult-born hippocampal neurons following traumatic brain injury
Source: PLoS One. 2020 Mar 2;15(3):e0229240. doi: 10.1371/journal.pone.0229240 (PMC7051085; doi:10.1371/journal.pone.0229240)
Supplement: S1 Table — DF = Degrees of Freedom; iGCL = Inner Granule Cell Layer, oGCL = Outer Granule Cell Layer, in the ipsilateral dentate gyrus; WT = Wild Type. KO = Knockout. The Sholl Analysis represents the average number of intersections in each condition (Column) function of the distance to the soma (Row). (PDF) [file pone.0229240.s003.pdf]

S3 Table. Summary of statistics for intra-genotype comparisons. DF = Degrees of Freedom; iGCL = Inner Granule Cell Layer, oGCL = Outer Granule Cell Layer, in the ipsilateral dentate gyrus; WT = Wild Type. KO = Knockout. The Sholl Analysis represents the average number of intersections in each condition (Column) function of the distance to the soma (Row).

Table Analyzed	Condition	Statistical Test	*p* value	DF	R^2	Statistic
1st Branch	WT	Kruskal-Wallis	<0.0001			H (3) = 18.44
	WT: iGCL vs oGCL	Dunn's test	<0.0001			
	WT: iGCL vs Contralateral	Dunn's test	0.5977			
	WT: oGCL vs Contralateral	Dunn's test	0.0073			
	ApoE KO	Kruskal-Wallis	<0.0001			H (3) = 26.38
	ApoE KO: iGCL vs oGCL	Dunn's test	<0.0001			
	ApoE KO: iGCL vs Contralateral	Dunn's test	0.6996			
	ApoE KO: oGCL vs Contralateral	Dunn's test	<0.0001			
	ApoE3	Kruskal-Wallis	0.004			H (3) = 11.05
	ApoE3: iGCL vs oGCL	Dunn's test	0.0028			
	ApoE3: iGCL vs Contralateral	Dunn's test	0.5781			
	ApoE3: oGCL vs Contralateral	Dunn's test	0.107			
	ApoE4	Kruskal-Wallis	<0.0001			H (3) = 21.26
	ApoE4: iGCL vs oGCL	Dunn's test	<0.0001			
	ApoE4: iGCL vs Contralateral	Dunn's test	0.2173			
	ApoE4: oGCL vs Contralateral	Dunn's test	0.0117			
Nodes	WT	Kruskal-Wallis	0.5029			H (3) = 1.375
	WT: iGCL vs oGCL	Dunn's test	0.8527			
	WT: iGCL vs Contralateral	Dunn's test	>0.9999			
	WT: oGCL vs Contralateral	Dunn's test	0.9441			
	ApoE KO	Kruskal-Wallis	0.2523			H (3) = 2.754
	ApoE KO: iGCL vs oGCL	Dunn's test	0.5608			
	ApoE KO: iGCL vs Contralateral	Dunn's test	0.2974			
	ApoE KO: oGCL vs Contralateral	Dunn's test	0.1043			
	ApoE3	Kruskal-Wallis	0.6295			H (3) = 0.9257
	ApoE3: iGCL vs oGCL	Dunn's test	>0.9999			
	ApoE3: iGCL vs Contralateral	Dunn's test	>0.9999			
	ApoE3: oGCL vs Contralateral	Dunn's test	>0.9999			
	ApoE4	Kruskal-Wallis	0.1277			H (3) = 4.116
	ApoE4: iGCL vs oGCL	Dunn's test	0.1914			
	ApoE4: iGCL vs Contralateral	Dunn's test	>0.9999			
	ApoE4: oGCL vs Contralateral	Dunn's test	0.2327			
Total Length	WT	Kruskal-Wallis	0.1369			H (3) = 0.1369
	WT: iGCL vs oGCL	Dunn's test	0.187			

	WT: iGCL vs Contralateral	Dunn's test	>0.9999		
	WT: oGCL vs Contralateral	Dunn's test	0.2969		
	ApoE KO	Kruskal-Wallis	0.0016		H (3) = 12.92
	ApoE KO: iGCL vs oGCL	Dunn's test	0.0014		
	ApoE KO: iGCL vs Contralateral	Dunn's test	0.8471		
	ApoE KO: oGCL vs Contralateral	Dunn's test	0.0019		
	ApoE3	Kruskal-Wallis	0.1239		H (3) = 0.1239
	ApoE3: iGCL vs oGCL	Dunn's test	0.1563		
	ApoE3: iGCL vs Contralateral	Dunn's test	0.0497		
	ApoE3: oGCL vs Contralateral	Dunn's test	0.6817		
	ApoE4	Kruskal-Wallis	0.0055		H (3) = 10.42
	ApoE4: iGCL vs oGCL	Dunn's test	0.1009		
	ApoE4: iGCL vs Contralateral	Dunn's test	0.5863		
	ApoE4: oGCL vs Contralateral	Dunn's test	0.0039		
Angle	WT	Kruskal-Wallis	0.0157		H (3) = 0.0157
	WT: iGCL vs oGCL	Dunn's test	0.0134		
	WT: iGCL vs Contralateral	Dunn's test	0.2533		
	WT: oGCL vs Contralateral	Dunn's test	0.6427		
	ApoE KO	Kruskal-Wallis	0.048		H (3) = 6.075
	ApoE KO: iGCL vs oGCL	Dunn's test	0.015		
	ApoE KO: iGCL vs Contralateral	Dunn's test	0.371		
	ApoE KO: oGCL vs Contralateral	Dunn's test	0.1016		
	ApoE3	Kruskal-Wallis	0.0157		H (3) = 8.303
	ApoE3: iGCL vs oGCL	Dunn's test	0.0149		
	ApoE3: iGCL vs Contralateral	Dunn's test	0.1807		
	ApoE3: oGCL vs Contralateral	Dunn's test	0.8697		
	ApoE4	Kruskal-Wallis	0.0008		H (3) = 14.34
	ApoE4: iGCL vs oGCL	Dunn's test	0.0005		
	ApoE4: iGCL vs Contralateral	Dunn's test	0.4125		
	ApoE4: oGCL vs Contralateral	Dunn's test	0.0546		
Sholl Analysis	WT: iGCL vs oGCL vs Contralateral	Two-way ANOVA			
		Interaction	<0.0001	56	F (56, 4727) = 2.651
		Row Factor	<0.0001	28	F (28, 4727) = 220.5
		Column Factor	<0.0001	2	F (2, 4727) = 12.95
	WT: iGCL vs Contralateral	Fisher's LSD	p < 0.05, 0.05, 0.01 for each 10µm increment between 30 and 50µm from the soma, p < 0.05 at 110µm from the soma, p < 0.05, 0.01, 0.001 for each 10µm increment between 130 and 150µm from the soma		

WT: oGCL vs Contralateral	Fisher's LSD	p < 0.05, 0.05 at 110 and 120µm from the soma, p < 0.05, 0.05 at 190 and 200µm from the soma		
WT: iGCL vs oGCL	Fisher's LSD	p < 0.01, 0.01, 0. 01, 0.05 for each 10µm increment between 30µm and 60µm from the soma, p < 0.01, 0.001, 0.001, 0.001, 0.01, 0.001, 0.01, 0.001, 0.01, 0.01 for each 10µm increment between 100 and 190µm from the soma		
ApoE KO: iGCL vs oGCL vs Contralateral	Two-way ANOVA			
	Interaction	<0.0001	56	F (56, 6815) = 3.696
	Row Factor	<0.0001	28	F (28, 6815) = 249.7
	Column Factor	<0.0001	2	F (2, 6815) = 30.67
ApoE KO: iGCL vs Contralateral	Fisher's LSD	p < 0.01, 0.05 and 0.01 for each 10µm increment between 200µm and 220µm from the soma		
ApoE KO: oGCL vs Contralateral	Fisher's LSD	p < 0.001, 0.001, 0.01 for each 10µm increment between 40µm and 60µm from the soma; p < 0.01, 0.05, 0.05, 0.05, 0.05, 0.05, 0.01, 0.01, 0.001, 0.01 and 0.05 for each 10µm increment between 100µm and 210µm from the soma		
ApoE KO: iGCL vs oGCL	Fisher's LSD	p < 0.01, 0.01, 0.05 for each 10µm increment between 40µm and 60µm from the soma; p < 0.05, 0.05, 0.01, 0.001, 0.01, 0.001, 0.01, 0.001, 0.001, 0.001, 0.001, 0.001, 0.001, and 0.01 for each 10µm increment between 100µm and 230µm from the soma		
ApoE3: iGCL vs oGCL vs Contralateral	Two-way ANOVA			
	Interaction	<0.0001	54	F (54, 6188) = 3.038
	Row Factor	<0.0001	27	F (27, 6188) = 347.4
	Column Factor	<0.0001	2	F (2, 6188) = 24.98
ApoE3: iGCL vs Contralateral	Fisher's LSD	p < 0.05 at 30 and 50µm from the soma; p < 0.05 and 0.05 for each 10µm increment between 100µm and 110µm from the soma; p < 0.05, 0.01, 0.001, 0.001, 0.001, 0.001, 0.001, 0.001 and 0.05 for each 10µm increment between 130µm and 210µm from the soma		

	ApoE3: oGCL vs Contralateral	Fisher's LSD	p < 0.05 at 40µm from the soma		
	ApoE3: iGCL vs oGCL	Fisher's LSD	p < 0.001, 0.001, 0.001 and 0.01 for each 10µm increment between 30µm and 60µm from the soma; p < 0.05 and 0.05 for each 10µm increment between 110µm and 120µm from the soma; p < 0.05, 0.05, 0.01, 0.01, 0.001, 0.01, 0.01 and 0.05 for each 10µm increment between 140µm and 210µm from the soma		
	ApoE4: iGCL vs oGCL vs Contralateral	Two-way ANOVA			
		Interaction	<0.0001	54	F (54, 7084) = 4.115
		Row Factor	<0.0001	27	F (27, 7084) = 223.7
		Column Factor	<0.0001	2	F (2, 7084) = 30.83
	ApoE4: iGCL vs Contralateral	Fisher's LSD	p < 0.05 at 120µm from the soma		
	ApoE4: oGCL vs Contralateral	Fisher's LSD	p < 0.001 and 0.01 for each 10µm increment between 40µm and 50µm from the soma; p < 0.05, 0.05, 0.01, 0.01, 0.001, 0.001, 0.001, 0.001, 0.001, 0.001, 0.01 and 0.05 for each 10µm increment between 110µm and 220µm from the soma		
	ApoE4: iGCL vs oGCL	Fisher's LSD	p < 0.05, 0.001, 0.001 and 0.01 for each 10µm increment between 30µm and 60µm from the soma; p < 0.01, 0.01, 0.001, 0.001, 0.001, 0.001, 0.001, 0.01 and 0.05 for each 10µm increment between 150µm and 230µm from the soma		
